# Red cell distribution width predicts short- and long-term outcomes of acute congestive heart failure more effectively than hemoglobin

**DOI:** 10.3892/etm.2014.1755

**Published:** 2014-06-04

**Authors:** YUXIANG DAI, HAKUOH KONISHI, ATSUTOSHI TAKAGI, KATSUMI MIYAUCHI, HIROYUKI DAIDA

**Affiliations:** 1 Department of Cardiology, Juntendo University School of Medicine, Tokyo 113-8421, Japan; 2 Shanghai Institute of Cardiovascular Diseases, Zhongshan Hospital, Fudan University, Shanghai 200032, P.R. China

**Keywords:** heart failure, biomarker, prognosis, anemia, red blood cell distribution width

## Abstract

The present study compared short- and long-term prognostic values of red blood cell distribution width (RDW) with those of hemoglobin (Hgb) among patients with acute congestive heart failure (CHF) in a cardiac care unit. The cross-sectional study examined data from 521 patients with acute CHF who were admitted to a cardiac care unit and followed up for 24 months (median). Mean Hgb levels in patients who succumbed (DIH) or remained alive (AIH) were 11.0±1.8 and 11.8±2.6 g/l (P>0.05), respectively. Median values of RDW were 16.2% and 14.4%, respectively (P<0.0001). During the 24-month follow-up, mean levels of Hgb in groups with and without endpoints were 11.4±2.5 and 12.5±2.4 g/dl (P<0.0001), respectively. Median RDW values were 14.9 and 13.8%, respectively (P<0.0001). Logistic regression analysis showed that in-hospital mortality was significantly associated with RDW (P=0.044), New York Heart Association (NYHA) functional class IV (P=0.0037), estimated glomerular filtration rate (eGFR) (P=0.042) and C-reactive protein (P=0.0044), but not with Hgb (P=0.10). The multivariate Cox proportional hazard model selected RDW [hazard ratio (HR), 2.19; P<0.0001], left ventricular ejection fraction (HR 0.81, P=0.0016), age (10-year increase; HR 1.19, P=0.0017) and NYHA functional classes III/IV (HR 1.52, P=0.0029) as independent predictors of long-term outcomes after adjustment, but not Hgb (HR 1.01, P=0.86). Higher RDW values in acute CHF patients at admission were associated with worse short- and long-term outcomes and RDW values were more prognostically relevant than Hgb levels.

## Introduction

Congestive heart failure (CHF) is often accompanied by high mortality rates. Therefore, accurate risk stratification is critically important in identifying high-risk patients who may benefit from advanced treatment (1–4). A number of clinical variables and biological markers have been applied over the last decade in predictive models of survival for patients with CHF (5,6), including inflammatory cytokines (7,8) high-sensitivity C-reactive protein (CRP) (9) natriuretic peptides (10,11) neurohormones (12) and oxidative stress (13), all of which are useful for diagnosis and prognosis. However, these biomarkers are very expensive to analyze and cost effectiveness must be considered when including markers in predictive models.

Anemia is a common feature of patients with CHF and it has important implications for the prognosis and treatment of CHF (14–17). A cause-and-effect relationship between anemia and CHF has not been demonstrated. Red blood cell distribution width (RDW) is a measure of the size variability in the red blood cell population and it is calculated as the standard deviation (SD) of the distribution of RDW divided by the mean corpuscular volume. Several studies have confirmed that RDW predicts mortality in acute coronary syndrome (18,19) and heart failure (20–22). However, the predictive value of RDW for in-hospital, and long-term outcomes for acute CHF and acute CHF predictive value relative to hemoglobin (Hgb) and anemia remain unknown (23).

The present study therefore investigated whether RDW is useful for risk stratification in patients hospitalized with acute exacerbation of CHF and analyzed the prognostic value of RDW compared with that of Hgb levels and anemia status.

## Materials and methods

### Patients

Patients with acute onset of CHF were consecutively enrolled in an observational study between January 2007 and December 2009. All patients were hospitalized at the cardiac care unit (CCU) of Juntendo University Hospital (Tokyo, Japan). Demographic and clinical information, as well as age, gender, New York Heart Association (NYHA) functional class, history of cigarette smoking, ischemic heart disease, hypertension, hyperlipidemia, atrial fibrillation, diabetes mellitus, chronic renal failure, hemodialysis and medications taken upon admission were obtained from medical records. Body mass index was calculated from height and weight data. Cardiac structure and function was assessed by echocardiography using a hematology analyzer (HF-3000, Chengdu, China).

The patients were followed up by telephone, personal interviews and by reviewing electronic medical records. The endpoint for short-term outcomes was in-hospital mortality. Mortality from all causes and readmission to hospital with the onset of CHF were long-term endpoints. The end of the follow-up was June 2010. The current study was performed according to the ethics policies of Juntendo University Hospital and was approved by the internal review board. Informed consent was obtained from the patient.

### Laboratory measurements

Blood samples were obtained immediately upon admission to the CCU and analyzed as quickly as possible in the clinical chemistry laboratory. Anemia was defined as Hgb <12 g/dl in women and <13 g/dl in men, according to the World Health Organization (WHO) criteria. Renal function was evaluated as the estimated glomerular filtration rate (eGFR), calculated according to the simplified modification of diet in renal disease (MDRD) equation: eGFR (ml/min/1.73 m^2^) = 186 × [serum creatinine (Scr)]^−1.154^ × (age)^−0.203^ × (0.742 if female) where Scr is the serum creatinine level in mg/dl.

### Statistical analysis

Discrete variables were presented as frequency counts and ratios (%). Continuous variables were analyzed based on a normal distribution using the Shapiro-Wilk test. They were expressed as mean ± SD when normally distributed, or as medians with inter-quartile ranges (IQRs) if not. Proportions and means or medians were compared using the χ^2^ test, the two-tailed independent Student’s t-test, one-way analysis of variance (ANOVA) and the Wilcoxon or Kruskal-Wallis one-way ANOVA test. Univariable correlations were examined using the Pearson’s correlation coefficient in the context of normality, whereas correlations among non-normally distributed variables were examined using the Spearman’s rho test. Variables with a retention P-value of 0.10 in the univariate analysis were entered into a multivariable linear regression model. Variables with non-normal distributions were log-transformed prior to being entered into this model.

Independent predictors of short- and long-term outcomes were identified using the logistic regression and Cox proportional hazard models, respectively. Kaplan-Meier accumulated survival curves were constructed and log-rank values were calculated to assess their statistical significance.

All data were analyzed using JMP 8.0 (SAS Institute, Inc., Cary, NC, USA) and SPSS 18.0 (SPSS Inc., Chicago, IL, USA). P<0.05 was considered to indicate a statistically significant difference.

## Results

### Patient characteristics

The current study comprised 521 patients with acute onset CHF from a wide range of age groups [72 (64, 80) years]. The median (IQR) values of RDW and mean level of Hgb overall were 14.5 (13.6, 15.9)% and 11.7±2.5 g/dl, respectively. Based on the WHO criteria, 324 (62.2%) patients were diagnosed with anemia. Among the patients, 192 (36.9%), 200 (38.4%) and 129 (24.8%) were classified as NYHA functional classes II, III and IV, respectively. Table I shows the patient characteristics upon admission, grouped according to quartiles of RDW. Patients with a higher RDW significantly differed from the others in terms of age, Hgb, anemia, NYHA classification, left ventricular ejection fraction (LVEF), B-type natriuretic peptide (BNP), lipid profiles [specifically, total cholesterol (TC), low density lipoprotein cholesterol (LDL-C), high density lipoprotein cholesterol (HDL-C) and triglycerides (TG)] blood urea nitrogen (BUN), eGFR, CRP and loop diuretics at presentation.

### Predictors of RDW values

Univariate analysis showed that age, history of CRF, hemodialysis at presentation, history of diabetes mellitus, Hgb, anemia, NYHA classification, LVEF, BNP, TC, LDL-C, HDL-C, TG, BUN, eGFR and loop diuretics at presentation were significantly correlated with RDW (P<0.05, data not shown). However, further multivariate analysis revealed Hgb, BNP, eGFR and HDL-C as independent predictors of RDW (Table II).

### Prediction value of in-hospital mortality and long-term outcome

Thirty-three (6.3%) patients succumbed in hospital. After a median follow-up of 24 months (range, 6–42 months), 315 (64.5%) patients reached the endpoint of mortality or re-hospitalization.

**Figure 1:**
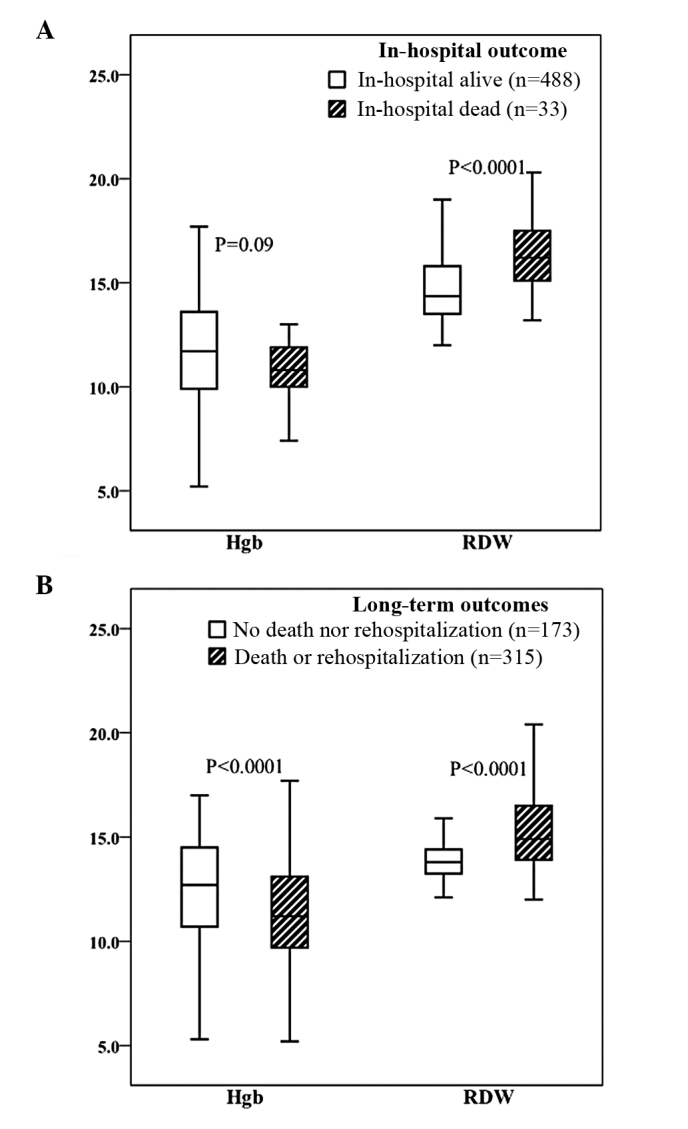
(A) Red blood cell distribution width was significantly higher in the group of patients who succumbed in hospital than in the group who remained alive in hospital [16.2 (15.1, 17.6)% vs. 14.4 (13.5, 15.8)%, P<0.001]. Hemoglobin did not differ significantly between the groups (11.0±1.8 vs. 11.8±2.6 g/dl, P>0.05). (B) Both hemoglobin and red blood cell distribution width significantly differed between the group that underwent no mortality or rehospitalization and the group that succumbed or underwent rehospitalization. Hgb, hemoglobin; RDW, red blood cell distribution width.

The RDW values were significantly higher in the group that succumbed while in hospital (DIH) compared with the group that remained alive while in hospital (AIH) [16.2 (15.1, 17.6)% vs. 14.4 (13.5, 15.8)%, P<0.0001)]. However, Hgb did not significantly differ between the two groups (11.0±1.8 vs. 11.8±2.6 g/dl, P>0.05; Fig. 1A). Logistic regression analysis showed that in-hospital mortality was significantly associated with RDW, NYHA (IV-III), eGFR and CRP (P<0.05), but not with Hgb (Table III).

Throughout the median 24 months of follow-up, both Hgb and RDW significantly differed between the two groups (Fig. 1B). The mean levels of Hgb in patients that reached an endpoint or did not reach an endpoint were 11.4±2.5 and 12.5±2.4 g/dl, respectively (P<0.0001); the median (IQR) values of RDW were 14.9 (13.9, 16.5)% and 13.8 (13.3, 14.4)% respectively (P<0.0001).

**Figure 2:**
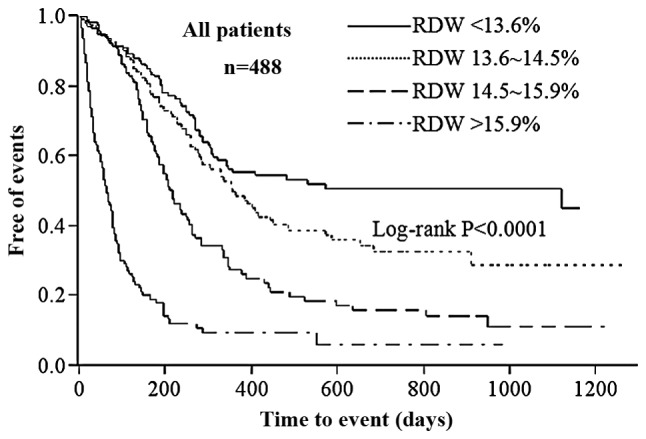
Kaplan-Meier survival curves for mortality rates from all causes and re-admission to hospital, stratified according to red blood cell distribuion width quartiles among all patients (n=488). Rates of reaching endpoints were significantly higher among those with higher red blood cell distribution widths (log-rank P<0.0001). RDW, red blood cell distribution width.

**Figure 3:**
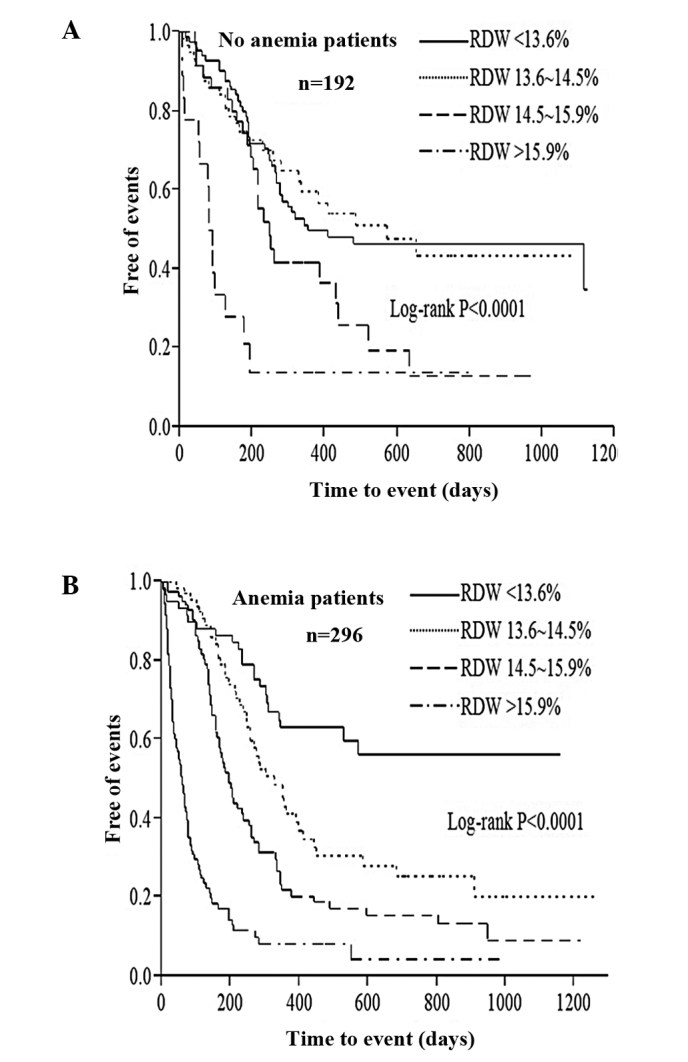
Kaplan-Meier survival curves for mortality rates from all causes and re-admission to hospital, stratified according to red blood cell distribution width quartiles in patients (A) without anemia and (B) with anemia. Rates of reaching endpoints were significantly higher among the patients without (n=192) and with (n=296) anemia who had higher red blood cell distribution widths. RDW, red blood cell distribution width.

**Figure 4:**
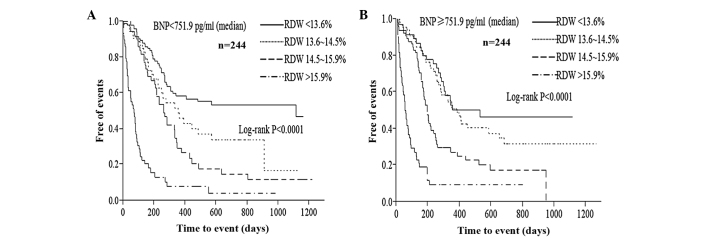
Kaplan-Meier survival curves for mortality rates from all causes and re-admission to hospital stratified according to red blood cell distribution width quartiles in patients with (A) lower and (B) higher B-type natriuretic peptide values. Lower (A) and higher (B) BNP values were < or ≥751.9 pg/ml, respectively. Medians were n=244 for each. BNP, B-type natriuretic peptide; RDW, red blood cell distribution width.

Kaplan-Meier survival curves for mortality from all causes and readmission to hospital demonstrated that rates of endpoints were significantly higher among patients with a higher RDW (Fig. 2; Log-rank P<0.0001). The graded increased probability of endpoints with increasing RDW quartiles during follow-up persisted regardless of the presence of anemia (Fig. 3) and variations in BNP level (Fig. 4). The univariate Cox proportional hazard model analysis (Table IV) associated Hgb [per SD increase, hazard ratio (HR), 0.72; 95% CI, 0.64–0.81; P<0.0001) and RDW (per SD increase, HR, 2.25; 95% CI, 2.02–2.49; P<0.0001] with higher risks of reaching endpoints. After adjustment in the multivariate Cox proportional hazard model analysis (Table IV), RDW remained a significant risk factor (per SD increase, HR, 2.19; 95% CI, 1.92–2.50; P<0.0001), whereas Hgb did not remain a predictive value (per SD increase, HR, 1.01; 95% CI, 0.96–1.13; P=0.86). In this final adjusted model, the other independent predictors included LVEF (per SD increase, HR, 0.81; 95% CI, 0.71–0.92; P=0.0016), age (10 years increase, HR, 1.19; 95% CI, 1.07–1.34; P=0.0017) and NYHA classes III and IV (HR, 1.52; 95% CI, 1.15–2.03; P=0.0029).

## Discussion

The results of the present study revealed that RDW may act as a powerful independent predictive factor for short- and long-term outcomes in patients with acute exacerbation of CHF and that this prognostic value remains significant even after adjustment for other known prognostic factors. The correlation between RDW and the long-term outcomes of patients with acute CHF provides prognostic data upon which to base risk factors and persists regardless of the levels of Hgb or BNP. Other prognostic markers identified in the current study were age, LVEF and NYHA class, all of which were consistent with the findings of published models on heart failure.

The RDW is a measurement of the size variation among circulating red cells and is calculated as part of the routine complete blood count (24). The RDW, along with mean cell volume (MCV), is useful in the differential diagnosis of the causes of anemia (25). The normal range for RDW is between 11.5% and 14.5%, and higher RDW values represent a greater variability in cell size (24,25). The median RDW value in the present study was 14.5%, indicating that half of the patients had an RDW value that was above the upper limit of the normal range.

Anemia has convincingly served as a powerful predictor of re-hospitalization rates and survival in CHF (14,26,27). Several mechanisms have been proposed to explain the association between anemia and outcomes of heart failure, including nutritional deficiencies, impaired renal function, inadequate production of erythropoietin, inflammatory stress (such as circulating cytokines and chemokines that predict a higher mortality risk in the population) and the impact of comorbidities (4,17,28). In addition, the hemodynamic changes accompanying severe anemia, including increased preload, reduced peripheral vascular resistance and increased cardiac output, may ultimately lead to a detrimental increase in left ventricular mass (15,27). Any or all of these mechanisms may also influence RDW.

The RDW values become elevated under conditions of increased red cell destruction or ineffective red cell production (24). Increased RDW may represent a nutritional deficiency (iron, vitamin B12 or folic acid), bone marrow depression or chronic inflammation (24,25). Inflammatory cytokines are predictors of the prognosis of heart failure. Proinflammatory cytokines inhibit erythropoietin-induced erythrocyte maturation, which is partly reflected by an increase in RDW (29). Therefore, RDW may serve as an integrative marker of multiple pathological processes that are involved in heart failure, including the above conditions, which are often found in patients with heart failure (30) This may explain why RDW values correlate with disease severity and are associated with prognosis. The current study identified Hgb, BNP, eGFR and HDL-C as independent predictors of RDW; eGFR reflected renal function and HDL-C reflected nutritional status. However, a significant relationship was not identified between RDW and inflammatory indices (CRP and UA) in the multivariate regression model.

The most notable finding of the present study was that after multivariate adjustment of all the patient data, RDW, but not Hgb, was predictive of both in-hospital and long-term outcomes. This indicates that the pathophysiology leading to increased RDW may affect outcomes in chronically ill patients, irrespective of anemia status. The finding may be explained as follows. As noted above, many variables are associated with anemia, such as inflammatory factors, nutritional deficiencies, renal dysfunction and inadequate erythropoietin production. The relationships among these variables and outcomes may be more directly reflected through their impact on RDW, than on Hgb. This was demonstrated in the present study as RDW predicted mortality in patients with and without anemia.

Secondly, RDW may be an earlier marker of prognosis than Hgb as it reflects the early steps in the complex processes that lead to anemia. At this point red blood cell production is ineffective and red blood cell destruction increases, but Hgb remains within the normal range. In addition, all the patients in the current study had the onset of acute CHF. Hemodynamic changes during this acute phase, such as fluid retention, interfere with the accurate measurement of Hgb, which may generate misinformation about the status of anemia. Under these circumstances, RDW is likely to be a more appropriate marker of baseline anemia status.

Another notable finding of the present study was that after stratifying levels of BNP, RDW remained associated with long-term outcomes. Although BNP is considered to be a powerful predictor of CHF outcomes (31), a single biomarker is insufficient to assess the outcomes of the entire study population due to disease heterogeneity. A recent multiple-marker approach has been suggested for risk stratification of heart failure (32–34). The current study found that a graded increased probability of endpoints with increased RDW during follow-up in groups with low and high BNP indicated that the predictive value of RDW was independent of that of BNP. Due to its widespread availability and cost-effectiveness, the inclusion of RDW in multiple marker models of CHF risk stratification is recommended.

The RDW is a readily available and inexpensive test for patients with CHF. Results are reported together with a complete differential blood count and no extra cost is incurred. The RDW has a better prognostic value than Hgb for both short- and long-term outcomes in patients with decompensated CHF and the prognostic value in long-term outcomes remains significant regardless of anemia or BNP levels. Thus, RDW appears to carry prognostic information concerning states other than anemia. In the future, the inclusion of RDW in a combined model for the risk stratification of patients with acute exacerbation of CHF is recommended. Further study is required to clarify the detailed mechanisms of the effect of elevated RDW in CHF.

## Figures and Tables

**Table I:** Baseline clinical characteristics of patients at time of admission, grouped according to red blood cell distribution width.

	RDW				
		
Characteristic	Quartile 1 (<13.6%, n=142)	Quartile 2 (13.6–14.5%, n=123)	Quartile 3 (14.5–15.9%, n=128)	Quartile 4 (>15.9%, n=128)	P-value
Demographics					
Age (years), median (IQR)	68 (62, 77)	74 (63, 81)	74 (66, 82)	73 (65, 80)	0.0012
Male	102 (71.8%)	79 (64.2%)	79 (61.7%)	87 (68.0%)	0.31
BMI (kg/m^2^), median (IQR)	21.2 (18.7, 24.7)	20.4 (18.4, 23.1)	20.5 (18.1, 23.5)	20.2 (17.8, 23.2)	0.39
Current smoker	8 (5.6%)	3 (2.4%)	6 (4.7)	6 (4.4%)	0.60
History of CRF	7 (4.9%)	11 (8.9%)	12 (9.4%)	17 (13.3)	0.11
Hemodialysis at presentation	3 (2.1%)	7 (5.7%)	7 (5.5%)	9 (7.0%)	0.23
History of hypertension	53 (37.3%)	56 (45.5%)	50 (39.1%)	42 (32.8%)	0.22
History of hyperlipidemia	18 (12.7%)	15 (12.2%)	12 (9.4%)	20 (15.6%)	0.51
History of atrial fibrillation	18 (12.7%)	21 (17.1%)	27 (21.1%)	25 (19.5%)	0.27
History of diabetes mellitus	14 (9.9%)	17 (13.8%)	22 (17.2%)	22 (17.2%)	0.24
Primary disease					0.20
Ischemic heart disease	48 (33.8%)	41 (33.3%)	45 (35.2%)	45 (35.2%)	
Hypertensive heart disease	36 (25.4%)	38 (30.9%)	26 (20.3%)	31 (24.2%)	
Valvular heart disease and congenital heart disease	14 (9.9%)	14 (11.4%)	25 (19.5%)	25 (19.5%)	
Dilated cardiomyopathy	17 (12.0%)	11 (8.9%)	10 (7.8%)	14 (10.9%)	
Hypertrophic cardiomyopathy	8 (5.6%)	7 (5.7%)	5 (3.9%)	6 (4.7%)	
Anemia					
Hemoglobin (g/dl), mean±SD	12.9±2.3	12.3±2.1	11.1±2.4	10.4±2.4	<0.0001
Anemia (World Health Organization)	60 (42.3%)	64 (52.0%)	91 (71.1%)	109 (85.2%)	<0.0001
Cardiac function					
NYHA					0.0049
Class II	71 (50.0%)	42 (34.2%)	41 (32.0%)	37 (29.1%)	
Class III	46 (32.4%)	54 (43.9%)	51 (39.8%)	49 (38.6%)	
Class IV	25 (17.6%)	27 (22.0%)	36 (28.1%)	41 (32.3%)	
LVEF (%), median (IQR)	50 (35, 58)	44 (35, 58)	43 (34, 54)	35 (28, 52)	0.0009
BNP (pg/ml), median (IQR)	444.0 (213.0, 1056.7)	822.6 (471.9, 1448.3)	834.1 (377.8, 1722.0)	917.6 (436.2, 1690.2)	<0.0001
Lipid and glucose profiles					
TC (mg/dl), median (IQR)	176 (150, 206)	164 (146, 196)	158 (135, 185)	157 (131, 188)	0.0060
LDL-C (mg/dl), median (IQR)	104 (74, 131)	101 (84, 123)	88 (73, 107)	87 (69, 112)	0.0038
HDL-C (mg/dl), median (IQR)	43 (36, 52)	45 (37, 51)	42 (35, 54)	38 (30, 45)	0.0005
TG (mg/dl), median (IQR)	91 (71, 134)	78 (60, 112)	82 (57, 115)	86 (63, 114)	0.05
HbA1c (%), median (IQR)	5.5 (5.1, 6.3)	5.5 (5.2, 6.1)	5.6 (5.3, 6.2)	5.6 (5.0, 6.4)	0.85
Renal function					
BUN (mg/dl), median (IQR)	19 (15, 27)	26 (17, 35)	29 (20, 43)	32 (21, 50)	<0.0001
eGFR, median (IQR)	85.0 (62.0, 99.2)	65.5 (33.0, 87.0)	53.6 (25.9, 80.5)	52.4 (26.9, 79.1)	<0.0001
Inflammation indices					
CRP (mg/dl), median (IQR)	0.6 (0.2, 3.5)	0.8 (0.2, 2.4)	1.0 (0.3, 3.8)	1.1 (0.4, 4.4)	0.03
Medications					
Digoxin at presentation	9 (6.4%)	8 (6.6%)	10 (7.8%)	16 (12.5%)	0.27
Thiazide at presentation	2 (1.4%)	2 (1.6%)	0 (0%)	4 (3.1%)	0.25
Loop diuretic at presentation	13 (9.2%)	15 (12.2%)	25 (19.5%)	26 (20.3%)	0.022
Aldosterone antagonist at presentation	11 (7.8%)	9 (7.3%)	13 (10.2%)	13 (10.2%)	0.77
β-blocker at presentation	23 (16.2%)	14 (11.4%)	24 (18.8%)	22 (17.2%)	0.40
ACE-I or ARB at presentation	22 (15.5%)	20 (16.3%)	22 (17.2%)	26 (20.3%)	0.75
Statins at presentation	15 (10.6%)	10 (8.2%)	10 (7.8%)	11 (8.6%)	0.86
Aspirin at presentation	23 (16.2%)	18 (14.6%)	30 (23.4%	24 (18.8)	0.29

SD, standard deviation; IQR, inter-quartile range; RDW, red blood cell distribution width; BMI, body mass index; CRF, chronic renal failure; NYHA, New York Heart Association; LVEF, left ventricular ejection fraction; BNP, B-type natriuretic peptide; TC, total cholesterol; LDL-C, low density lipoprotein cholesterol; HDL-C, high density lipoprotein cholesterol; TG, triglyceride; HbA1c, hemoglobin A1c; BUN, blood urea nitrogen; eGFR, estimated glomerular filtration rate; CRP, C-reactive protein.

**Table II:** Multiple linear regression assessing independent predictors of red blood cell distribution width.

Predictor	Estimated coefficient	P-value
Hgb	−0.27	<0.0001
Log BNP	0.39	0.0013
Log eGFR	−0.55	0.0096
Log HDL-C	−0.85	0.047
NYHA (IV-III)	0.53	0.059
Log BUN	0.40	0.18
Log LDL-C	−0.80	0.18
Loop diuretic at presentation	0.39	0.24
Log TC	0.86	0.35
NYHA (III-II)	0.20	0.44
Hemodialysis	−0.25	0.50
Log TG	−0.14	0.59
Log LVEF	−0.14	0.60
DM	−0.087	0.61
CRF	0.096	0.76
Log Age	0.18	0.79

Log, logarithmic transformed values used due to non-normal distribution; Hgb, hemoglobin; BNP, B-type natriuretic peptide; eGFR, estimated glomerular filtration rate; HDL-C, high density lipoprotein cholesterol; NYHA, New York Heart Association; BUN, blood urea nitrogen; LDL-C, low density lipoprotein cholesterol; TC, total cholesterol; TG, triglyceride; LVEF, left ventricular ejection fraction; DM, diabetes mellitus; CRF, chronic renal failure.

**Table III:** Logistic regression analysis for in-hospital mortality.

Predictor	Estimated Coefficient	P-value
Log RDW	5.21	0.044
Hgb	0.28	0.10
Log age	2.18	0.19
Gender (male)	0.61	0.090
Log BMI	−3.81	0.06
NYHA (III-II)	0.76	0.57
NYHA (IV-III)	2.56	0.0037
Log LVEF	−0.30	0.69
Log BNP	0.16	0.70
Log eGFR	−2.08	0.042
Log BUN	0.72	0.26
Log CRP	0.75	0.0044
Log UA	−0.89	0.23

Log, logarithmic transformed values used due to non-normal distribution; RDW, red blood cell distribution width; Hgb, hemoglobin; BMI, body mass index; NYHA, New York Heart Association; LVEF, left ventricular ejection fraction; BNP, B-type natriuretic peptide; eGFR, estimated glomerular filtration rate; BUN, blood urea nitrogen; CRP, C-reactive protein; UA, uric acid.

**Table IV:** Cox proportional hazard models for long-term outcomes.

	Univariate, unadjusted		Multivariate, adjusted	
		
Predictor	HR (95% CI)	P-value	HR (95% CI)	P-value
RDW (per SD increase)	2.25 (2.02–2.49)	<0.0001	2.19 (1.92–2.50)	<0.0001
Hgb (per SD increase)	0.72 (0.64–0.81)	<0.0001	1.01 (0.96–1.13)	0.86
LVEF (per SD increase)	0.71 (0.63–0.79)	<0.0001	0.81 (0.71–0.92)	0.0016
NYHA (III/IV)	2.01 (1.58–2.58)	<0.0001	1.52 (1.15–2.03)	0.0029
eGFR (per SD increase)	0.78 (0.69–0.87)	<0.0001	0.92 (0.78–1.07)	0.29
Age (10 years increase)	1.19 (1.08–1.30)	0.0002	1.19 (1.07–1.34)	0.0017
BUN (per SD increase)	1.20 (1.09–1.31)	0.0004	0.99 (0.85–1.14)	0.92
HDL-C (per SD increase)	0.87 (0.76–0.98)	0.0223	1.04 (0.96–1.13)	0.94
Log (CRP)	1.07 (1.00–1.15)	0.0448	1.04 (0.96–1.12)	0.34
History of diabetes mellitus	1.30 (0.96–1.72)	0.084	0.93 (0.66–1.29)	0.68

CI, confidence interval; HR, hazard ratio; RDW, red blood cell distribution width; Hgb, hemoglobin; LVEF, left ventricular ejection fraction; NYHA, New York Heart Association; eGFR, estimated glomerular filtration rate; BUN, blood urea nitrogen; HDL-C, high density lipoprotein cholesterol; Log, logarithmic transformed values used because of non-normal distribution.
